# The interplay of DAMPs, TLR4, and proinflammatory cytokines in pulmonary fibrosis

**DOI:** 10.1007/s00109-021-02113-y

**Published:** 2021-07-13

**Authors:** Siavash Bolourani, Max Brenner, Ping Wang

**Affiliations:** 1 Center for Immunology and Inflammation, Feinstein Institutes for Medical Research, 350 Community Dr, Manhasset, NY 11030 USA; 2 Elmezzi Graduate School of Molecular Medicine, Manhasset, NY USA; 3 Department of Surgery, Donald and Barbara Zucker School of Medicine At Hofstra/Northwell, Manhasset, NY USA; 4 Department of Molecular Medicine, Donald and Barbara Zucker School of Medicine At Hofstra/Northwell, Manhasset, NY USA

**Keywords:** DAMP, TLR4, Cytokine, Pulmonary fibrosis

## Abstract

Pulmonary fibrosis is a chronic debilitating condition characterized by progressive deposition of connective tissue, leading to a steady restriction of lung elasticity, a decline in lung function, and a median survival of 4.5 years. The leading causes of pulmonary fibrosis are inhalation of foreign particles (such as silicosis and pneumoconiosis), infections (such as post COVID-19), autoimmune diseases (such as systemic autoimmune diseases of the connective tissue), and idiopathic pulmonary fibrosis. The therapeutics currently available for pulmonary fibrosis only modestly slow the progression of the disease. This review is centered on the interplay of damage-associated molecular pattern (DAMP) molecules, Toll-like receptor 4 (TLR4), and inflammatory cytokines (such as TNF-α, IL-1β, and IL-17) as they contribute to the pathogenesis of pulmonary fibrosis, and the possible avenues to develop effective therapeutics that disrupt this interplay.

## Introduction

Pulmonary fibrosis is a chronic restrictive lung disease characterized by a progressive decline in lung volume capacity, resulting from many chronic inflammatory disorders affecting the lung [1–3]. The visibility of pulmonary fibrosis, in particular, has significantly increased during the 2020 COVID-19 pandemic [4, 5]. Most hospitalized patients with COVID-19 have bilateral interstitial pneumonitis, as indicated by ground-glass opacities [6], and many show signs of fibrosis with their lung capacity reduced by up to 30% [7, 8]. In addition to infections such as COVID-19, pulmonary fibrosis can also occur in the contexts of repeated inhalation of foreign particles (such as silicosis and pneumoconiosis) and autoimmune diseases (such as systemic autoimmune diseases of the connective tissue) [9, 10]. The prototypical form of chronic fibrotic condition of the lung, however, is idiopathic pulmonary fibrosis (IPF), for which only pirfenidone (Esbriet, Genentech) [11] and nintedanib (Ofev, Boehringer Ingelheim) [12] have been FDA-approved to attenuate the rate of disease progression. IPF’s median survival from diagnosis is 4.5 years [13], underlining the urgent medical need for more effective therapeutic approaches. Multiple genome-wide association studies (GWAS) have reported genetic association signals in patients with IPF, stressing the importance of host defense, cell–cell adhesions, and DNA repair in the pathogenesis of the disease [14–18]. Furthermore, the altered host defense mechanisms explain not only the possible triggering of pulmonary fibrosis by chronic inflammation and viral infection but also the susceptibility of pulmonary fibrosis patients to viral-induced exacerbations [19].

During the past 10 years, damage-associated molecular pattern (DAMP) molecules have been shown to play a vital role in promoting exacerbation, remodeling, and silent progression of pulmonary fibrosis [20]. Toll-like receptors (TLRs), by virtue of being pattern recognition receptors of DAMPs, have been identified as critical mediators through which DAMPs exert their effect in cellular microenvironments. It is now clear that inflammation, though not the only trigger of fibrosis, plays a key role in the activation of fibroblasts — a cellular process critical in the development of pulmonary fibrosis. The pathogenetic model that we present in this review focuses on how DAMP signaling at the cellular level tilts the scale from remodeling and fibrosis resolution towards self-perpetuating cycles of connective tissue deposition leading to clinically relevant fibrosis.

## DAMPs and TLR4 in pulmonary fibrosis

Intermittent episodes of transient inflammation in the lungs triggered by pathogens, chemical irritants, or autoimmunity can result in the necrosis and apoptosis of the epithelial cells and cause the release of intracellular components that act as DAMPs. The released DAMPs then activate homeostatic processes that most often promote the resolution of the insult underlying the inflammatory process. However, during more prolonged pathological states, this process is exaggerated, turning the homeostatic pulmonary environment into a self-perpetuating cycle of inflammation and DAMP release, resulting in pulmonary fibrosis.

Multiple structurally diverse DAMPs have been identified to act as mediators for this vicious cycle [20]. These include intracellular peptides [21], glycoproteins [22, 23], phospholipids [24], and even nucleic acids [25, 26] that are released to the environment during cell injury and necrosis processes which drive progressive tissue fibrosis. Once released, these endogenous ligands exert their effect mainly through TLRs [22]. TLRs are pattern recognition receptors to which DAMPs bind and, with the help of adaptor proteins, activate intracellular signal transduction cascades eliciting changes in gene expression and altering various cellular activities. Here, we focus on the profibrotic role of TLR4.

Among the TLRs, TLR4 has been shown to have a profibrotic effect in the lung when stimulated by DAMPs [27]. The first series of publications that illuminated the role of the TLR4 pathway on fibroblasts showed the activation of TLR4 enhances the process of fibrosis in the liver by downregulation the transforming growth factor (TGF)-β pseudoreceptor Bambi through TLR4 → MyD88 → NF-κB pathway, which causes sensitization of hepatic stellate cells (HSCs) to TGF-β1–induced signals and allows unrestricted activation of HSCs and differentiation to extracellular matrix (ECM)-producing myofibroblasts [28]. In this pioneering work, TLR4 was stimulated using lipopolysaccharide (LPS), a well-known and highly sensitive TLR4 activator [29–31]. Almost 11 years later, a similar effect was observed in persistent fibrosis of the lung through TLR4/myeloid differentiation 2 (MD2) complex related pathways and activation of pulmonary fibroblasts to myofibroblasts [32]. The stimulatory molecules used by Bhattacharyya et al. were tenascin-C (a multifunctional hexameric ECM protein) and fibronectin-extra domain A (Fn-EDA), which are potent TLR4 agonists generated within the injured pulmonary extracellular microenvironments [33–37]. The role of TLR4-activating DAMPs in pulmonary fibrosis has been further evaluated with high-mobility group box1 (HMGB1), a potent inducer of TLR4 [38] in pulmonary fibrosis. HMGB1 is highly expressed in IPF lungs, and its blockade with antibodies attenuates bleomycin-induced fibrosis [39]. Along the same line is the small heat shock protein alphaB-crystallin (HSPB5), implicated in the TLR4-dependent induction and progression of pulmonary fibrosis [40, 41]. Mice deficient in HSPB5 had an attenuated response to bleomycin-induced pulmonary fibrosis [42]. Another category of TLR4 agonists that have recently been identified to be involved in the progression of pulmonary fibrosis consists of S100 proteins. Higher levels of S100A4 have been shown to independently correlate with worse disease progression in IPF [43], and S100A4 has been shown to contribute to fibrosis by activating pulmonary fibroblasts [44] (Table 1).

**Table 1:** TLR4 stimulating DAMPs described in this review, their size, functionality, and primary location

Name	Size	Functionality	Primary location
HMGB1	215 amino acids	Chromatin-binding	Nucleus
S100A4	121 amino acids	Calcium-binding	Nucleus/cytoplasm
HSPB5	44 amino acids	Protein chaperone	Nucleus
CIRP	192 amino acids	RNA-binding	Nucleus
Fn-EDA	500 kDa glycoprotein	Collagen-binding	Extracellular matrix
Tenascin-C	2,000 kDa glycoprotein	Cell–cell signaling	Extracellular matrix

The induction of DAMPs following tissue injury or cell death in chronic inflammatory diseases has been studied extensively [45]. Oxidative stress and ECM matrix stiffness can also damage the microenvironment and contribute to the cycle of sustained fibrosis by the release of DAMPs [46]. However, support for whether this induction happens by direct effects on macrophages or fibroblasts to release DAMPs in the microenvironment is still lacking. Although some studies have suggested HMGB1 can be induced by reactive oxygen species (ROS) in macrophage and fibroblasts [47, 48], there is no evidence that HSPB5 protein can be induced by ROS in macrophages [49]. Furthermore, there is no conclusive evidence on whether the direct effect of ROS induces any profibrotic DAMPs in macrophages or fibroblasts in the fibrotic microenvironment. While Fn-EDA and tenascin-C are extracellular DAMPs contributing to ECM stiffness implicated in pulmonary fibrosis [35, 50], we know of no study that investigated the relationship between ECM stiffness and induction of profibrotic DAMPs at the cellular level in macrophages or fibroblasts.

## Inflammatory cytokines and pulmonary fibrosis

Cytokines are proteins involved in cell signaling, including interferons, interleukins, tumor necrosis factors, and chemokines. Over the past 10 years, much evidence has been accumulated in the role of proinflammatory cytokines in fibrogenesis and myofibroblast differentiation [51, 52]. Cytokines that did not use to be part of the discussion in pulmonary fibrosis have recently been shown to be integral to several pathways that drive pulmonary fibrosis [53–57]. The overarching mechanisms by which proinflammatory cytokines tip the scale towards fibrogenesis include the recruitment of immune cells, regulation of the fibroblast activation status, and production of other profibrotic cytokines, among which is TGF-β1, the master regulator of fibrosis.

Proinflammatory cytokines can be regulated in pulmonary fibrosis by oxidation stress and redox signaling through induction of mitochondria-derived ROS [58–60], NADPH oxidase (NOX) [61–65], and antioxidant depletion [60, 66–68]. They can also be regulated by ECM matrix stiffness through deposition of collagen [69] and cross-linking 224 875 5689 with fibronectin [70] in the fibrotic tissue microenvironment. As discussed later, evidence shows that these cytokines can also be induced by DAMP stimulation of macrophages and fibroblasts. Regardless of how they are induced, proinflammatory cytokines have shown to be a profibrotic player in the early phase of fibrosis [51]. At the cellular level, these cytokines exert their effect by three mechanisms: directly inducing fibroblast activation, causing the release of profibrotic cytokines (including TGF-β1) in immune cells/fibroblasts, or promoting the persistent autocrine/paracrine activation of fibroblasts. Among the most studied proinflammatory and profibrotic cytokines is tumor necrosis factor-alpha (TNF-α), interleukin (IL)-1β, and IL-17.

### TNF-α

In the case of TNF-α, all three cellular mechanisms of fibrosis have been described [71–75]. The profibrotic effect of TNF-α can be seen in the lungs of patients with IPF expressing high levels of TNF-α [76]. TNF-α released from M1 macrophages (classically activated macrophages, involved in secretion of proinflammatory cytokines) not only changes the phenotype of other macrophages and fibroblasts from reparative to inflammatory and delay tissue repair [77, 78] but also induces the release of TGF-β1 and platelet-derived growth factor (PDGF) from fibroblasts which in turn mediate fibroblast activation and production [79, 80]. Furthermore, even quiescent fibroblasts, which are resistant to activation by TLR agonists, will respond to TNF-α [81, 82]. TNF-α stimulated fibroblasts to secrete lumican and express integrins that promote persistent activation of fibroblast in an autocrine and paracrine fashion [83–85]. Less is known, however, about the release of TNF-α in the fibrotic microenvironment. ROS intermediates regulate the release of TNF-α from macrophages and fibroblasts [86], and NOX generated ROS participate in TNF-α-induced expression of vascular cell adhesion molecule 1 (VCAM-1) [87], which is a cell adhesion molecule highly expressed in the lungs of IPF patients [88] that is required for fibroblast activation [89]. The role of ECM stiffness in the release of TNF-α in a cellular fibrotic microenvironment is less clear. ECM stiffness has been shown to increase the release of TNF-α from RAW 264.7 murine macrophages [90]. However, the release of TNF-α was inversely proportional to ECM stiffness in THP-1 human macrophages [91]. Further studies are required to determine the effect of ECM stiffness and TNF-α release in the fibrotic pulmonary microenvironment.

### IL-17

Like TNF-α, IL-17 has been shown to play an important role in pulmonary fibrosis. Higher levels of IL-17 are found in lung tissues of IPF patients [92]. The mechanisms by which IL-17 is involved in the induction of fibrosis are likely very similar to those of TNF-α [93–95]. Furthermore, TNF-α and IL-17 have been shown to be the leading players in the recruitment of immune cells in the early stages of fibrosis [96]. The combination of these effects means that, overall, TNF-α and IL-17 are involved in sustained and intense activation of fibroblasts [97]. However, evidence has emerged that the effects of IL-17 on pulmonary fibrosis may be temporally distinct from those of TNF-α. While IL-17 has been shown to enhance the proliferation of fibroblasts [98], collagen deposition does not increase in the presence of IL-17 [99], and in fact, the signaling pathway of IL-17 is downregulated during collagen deposition [100]. Nevertheless, the precise role of IL-17 in fibroblast activation remains to be elucidated. The role of oxidative stress in the production of IL-17 has also remained unclear. While ROS induce TNF-α expression in macrophages and fibroblasts [87, 101] and aid IL-17 induced proliferation of fibroblasts [102], they have not been shown to increase the expression of IL-17 directly. Furthermore, to our knowledge, no study has yet shown the correlation between ECM stiffness and IL-17 expression on macrophages.

### IL-1β

The profibrotic role of IL-1β has long been known: mice overexpressing IL-1β have an exacerbated response to bleomycin-induced lung fibrosis [103]. Like TNF-α, IL-1β is a potent proinflammatory cytokine that induces activation of fibroblasts via the release of profibrotic cytokines like TGF-β1 [104]. Multiple pathways have been studied in connection with the direct effect of IL-1β on fibroblast activation [105–107]. Some studies have suggested that IL-1β is a cytokine upstream of IL-17 or that the profibrotic effect of IL-1β is contingent on IL-17 [108–110]. Other studies have indicated that the profibrotic effects of IL-1β are mediated through the IL-1 receptor 1 (IL-1R1)/myeloid differentiation primary response 88 (MyD88) pathway [111, 112]. Further studies are needed to elucidate the exact mechanism by which IL-1β tilts the immune cells and fibroblasts towards persistent fibrosis in the lung microenvironment.

## Connecting DAMPs, TLR4, and proinflammatory cytokines

In the previous sections, we reviewed the profibrotic effects of individual DAMPs and proinflammatory cytokines in the development of fibrosis. However, it should be noted that the interplay between DAMPs and cytokines exerts a critical role in the development and sustainment of fibrosis. The interaction between DAMPs and TLR4 causes the release of numerous proinflammatory cytokines on macrophages and fibroblasts [113, 114]. These cytokines can, in turn, activate other macrophages and fibroblasts, as described in the previous section. This interplay has been demonstrated by induction of TNF-α and IL-1β expression in fibroblasts by activating the TLR4 pathway [115] using LPS. HMGB1 has also been shown to induce TNF-α and IL-1β signaling in macrophages through the TLR4-dependent pathway [85, 116, 117]. Similarly, HSPB5 has been shown to increase IL-1β and the nuclear localization of Smad4 [42, 118], which is likely enhanced by TLR4 signaling [118].

One built-in defense mechanism against the development of pathological fibrosis is the induction of negative feedback loops by cytokines and DAMPs. TGF-β1 and IL-10 released by inflammatory macrophages and fibroblasts, for example, are potent inhibitors of inflammation in macrophages and fibroblasts which can tilt the organ towards resolution of fibrosis [119–121] in the late phase of fibrosis [122]. Furthermore, DAMPs can be protective against or involved in the resolution of fibrosis in some TLR signaling pathways. While fibroblast-specific deficiency of TLR4 has been shown to be protective against fibrosis, and TLR2 has shown to exacerbate bleomycin-induced pulmonary fibrosis by inducing an oxidative response [123–125], mice deficient in both TLR4 and TLR2 have been shown to have increased pulmonary fibrosis in response to radiation injury [126–128]. There are also antifibrotic TLRs that contrast the effect of DAMPs on profibrotic TLRs [22, 129]. TLR3 has been shown to have an antifibrotic effect by downregulation of the TGF-β1 signaling pathway and autocrine induction of interferon (IFN)-β [130–132]. Moreover, TLR3 deficiency in fibroblasts has also been shown to increase collagen deposition and profibrotic cytokines, suggesting the role of DAMPs through TLR3 in the resolution of fibrosis [133]. Similarly, TLR9-mediated IFN-β induction in fibroblasts has shown to be protective against pulmonary fibrosis, and TLR9-deficient mice have exacerbated pulmonary fibrosis [134].

When taken together, a picture emerges that juxtaposes the interaction of DAMPs and cytokines through TLR4 promoting persistent fibrosis and, through other TLRs, the resolution of fibrosis. The pathology ensues when the balance is tilted towards the persistent profibrotic pathway by different sections of the pathway perpetuated through positive feedback. This has therapeutic potential in fibrotic diseases of the lung not only by disrupting TLR4 pathways and DAMPs but also by inducing antifibrotic TLRs.

## Therapeutic considerations

While the research in therapeutic approaches to pulmonary fibrosis is ongoing, treatment strategies targeting the DAMPs, TLR4, and proinflammatory cytokines pathway have shown promising results in preclinical models (Table 2). Anti-HMGB1 antibody significantly attenuated lung fibrosis in a mouse model [39]. In addition, there is evidence that inhibition of HMGB1 will diminish fibroblast activation [135] and can disrupt the process of fibrosis [136]. Furthermore, silencing HMGB1 or its downstream signaling has proven successful in inhibiting the fibrotic process in different conditions [137, 138]. Anti-S100A4 has been shown to prevent bleomycin-induced pulmonary fibrosis in mice [44]. While the effect of anti-HSPB5 antibody in pulmonary fibrosis has not been studied, HSPB5-deficient mice have attenuated pulmonary fibrosis in response to bleomycin [42]. Among the extracellular TLR4 agonists present in the pulmonary fibrotic microenvironment, neutralizing tenascin-C is a promising target for antifibrotic therapy. Not only do tenascin-C-deficient mice have an attenuated response to bleomycin-induced lung fibrosis, but this process has also been shown to be TLR4 dependent [35].

**Table 2:** Potential molecular targets, therapeutic, and the stage of investigation for the interplay described in this review

Molecular target	Potential therapeutic	Stage of investigation	References
HMGB1	Anti-HMGB1 antibody	Mouse models have shown attenuated response to fibrosis	[39, 136–138]
S100A4	Anti-S100A4 antibody: 3B11^*^	Mouse models have shown attenuated response to fibrosis	[42, 44]
HSPB5	Anti-HSPB5 antibody	HSPB5-deficient mice have attenuated fibrotic response	[42, 44]
Tenascin-C	Anti-tenascin-C antibody: ST2485^*^	Mouse models have shown attenuated response to fibrosis, and the process is TLR4 dependent	[33–35]
TLR4/MD2	Anti-TLR4/MD2 complex antibody: T5342126	Mouse models have shown attenuated response to fibrosis	[32–35]
TNF-α	Anti-TNF-α antibody: etanercept	Two double-blind randomized control trials have shown reduced disease progression	[11, 140–145]
IL-1β	Anti-IL-1β antibody: canakinumab^*^	IL-1R1 deficiency in mice and monoclonal antibody has been shown to attenuate fibrosis in mice	[111, 146]
IL-17	Anti-IL-17 antibody: secukinumab^*^, brodalumab^*^, and ixekizumab^*^	Mouse models have shown attenuated response to fibrosis	[93, 147]

^*^These agents have not yet been studied in the context of fibrosis

While studies have looked at the effect of anti-TLR4 in stopping pulmonary fibrosis, many have failed. This is due to the fact that while TLR4 drives persistent fibrosis and fibroblast activation, TLR4 is also required for the resolution of fibrosis [139]. However, there is a promise that specifically targeting specific TLR4/MD2 signaling complexes, which are responsible for the profibrotic effect of TLR4, can provide potential therapeutic strategies [32, 35].

Anti-TNF-α antibodies embody the most successful therapeutic approaches to fibrotic lung diseases. While multiple studies have shown the therapeutic effects in animal models [140–142], a double-blind clinical trial of IPF patients treated with etanercept, a monoclonal antibody against TNF-α, improved neither the forced vital capacity nor the diffusing capacity of the lungs. However, it showed a non-significant improvement in function and quality of life measures [143]. The multicentric double-blind clinical trial “A Study of Cardiovascular Events in Diabetes” (ASCEND) showed that pirfenidone, a non-peptide synthetic molecule with anti-TNF-α activity, reduced disease progression in patients with IPF [11]. Additionally, a study combining the results of two previous trials of pirfenidone in IPF patients [144] observed a significant decrease in the risk of death after treatment [145]. While there has not been a trial evaluating the effect of neutralizing IL-1β in IPF, mice deficient in IL-1R1 are protected and developed attenuated bleomycin-induced pulmonary fibrosis [111]. Moreover, a monoclonal anti-IL-1β antibody has also been shown to attenuate silica-induced fibrosis in mice [146]. Along the same line, blocking IL-17 has shown to attenuate pulmonary fibrosis in both silica and bleomycin-induced pulmonary fibrosis models in mice and to promote resolution of fibrosis [93, 147].

## Conclusion and perspective

**Fig. 1:**
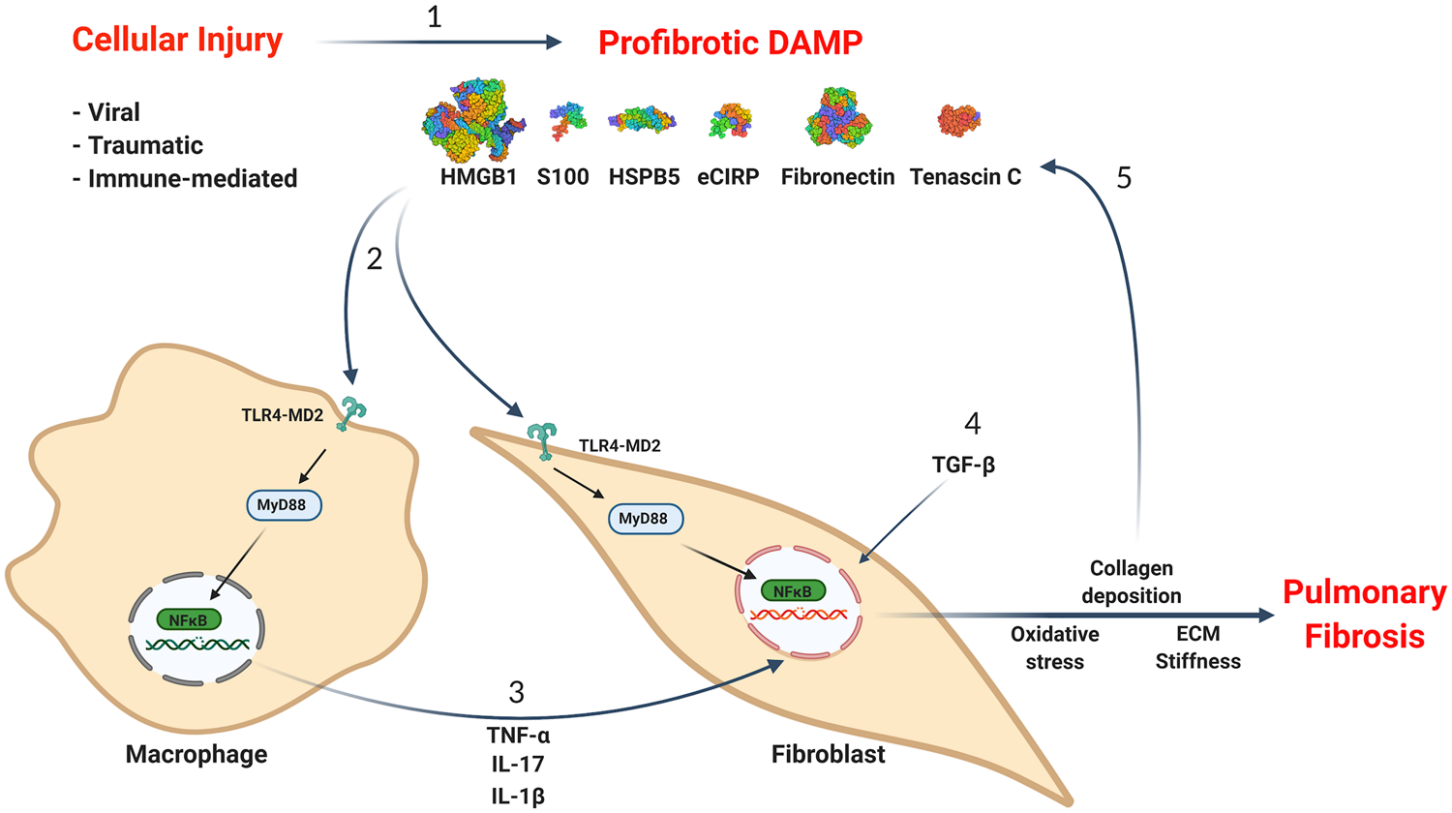
The interplay of DAMPs, TLR4, and proinflammatory cytokines in pulmonary fibrosis centered around macrophages and fibroblasts. (1) Injury to the cells either from a viral infection, chemical/mechanical trauma, or immune-mediated damage causes the release of DAMPs in the microenvironment. (2) DAMPs stimulate and activate macrophages and fibroblasts through a TLR4-MD2 → MyD88-mediated pathway. (3) Activated macrophages release proinflammatory cytokines such as TNF-α, IL-17, and IL-1β in the tissue microenvironment that, (4) along with TGF-β, activate fibroblasts to become profibrotic and deposit collagen and ECM components like fibronectin and tenascin-C. This causes stiffness of ECM and oxidative stress in the microenvironment, which (5) causes the release of more DAMPs leading to the vicious cycle of pulmonary fibrosis. DAMP, damage-associated molecular patterns; HMGB1, high-mobility group box 1; eCIRP, extracellular cold-inducible RNA-binding protein; HSPB5, heat shock protein B5; TLR4, Toll-like-receptor 4; MD2, myeloid differentiation factor 2; MyD88, myeloid differentiation primary response 88; NF-κB, nuclear factor kappa-light-chain-enhancer of activated B cells; ECM, extracellular matrix

Strong evidence has emerged that pulmonary fibrosis results from a cycle receiving positive feedback at multiple checkpoints that are instigated by DAMP induction of proinflammatory cytokines through TLR4 receptors. The process starts with an injury either from a viral infection, chemical/mechanical trauma, or immune-mediated damage that causes the release of DAMPs in the microenvironment (Fig. 1). The DAMPs then reprogram resident macrophages and fibroblasts towards a proinflammatory/profibrotic phenotype in a TLR4-dependent process. This prompts the deposition of extracellular collagen leading to ECM stiffness and the further release of DAMPs and proinflammatory/profibrotic cytokines along with the secretion of TGF-β1, the master regulator of fibrosis. TGF-β1, in turn, causes autocrine/paracrine activation of other macrophages and fibroblasts in the microenvironment that feeds the vicious cycle of persistent fibrosis. In our not yet published observations, we have discovered that extracellular cold-inducible RNA-binding protein (eCIRP), a DAMP that causes inflammation and organ injury in sepsis, hemorrhagic shock, and ischemia/reperfusion injury [148, 149], also plays an important role in the pathogenesis of pulmonary fibrosis. By targeting eCIRP, we may be able to ameliorate the fibrotic process in the lungs.

In this review, we have focused on a selected number of inflammatory cytokines, namely TNF-α, IL-1β, and IL-17, and showed the interplay of TLR4, DAMPs, and these cytokines. There are, however, other cytokines and chemokines that have shown to be either involved in this interplay or contribute to processes occurring in ECM, such as the production of ROS, which can contribute to the cycle of persistent fibrosis in the lung. In the class of interleukins alone, IL-2, IL-6, IL-9, IL-12, IL-13, and IL-27 have critical roles in the regulation of pulmonary fibrosis [150–156]. Among these, IL-6’s contributing mechanisms to the fibrotic process are likely very similar to those of TNF-α and IL-17 [157]. Just like TNF-α, IL-6 is released by M1 macrophages and changes the phenotype of other macrophages and fibroblasts from reparative to inflammatory and delays tissue repair [158]. Some studies suggest that blocking IL-6 can have the opposite effect on lung fibrosis [159]. This effect is due to the protective effect of IL-6/Stat3 signaling axes in alveolar epithelial cells against apoptosis, which are imperative for the production of surfactant synthesis necessary for the protection of the lung during injury [160]. Therefore, the timing of the anti-IL-6 strategy in the treatment of lung injury is crucial in antifibrotic therapeutic approaches [161]. While IL-6 is one of the most studied inflammatory cytokines in pulmonary fibrosis, its precise role in regulating the process of fibrosis in inflammatory diseases of the lung remains to be elucidated.

Additionally, we focused on the fibrotic effect of TLR4 in the early phases of fibrosis in this review. However, as mentioned earlier in the review, TLR4 also plays a crucial role in the resolution of fibrosis in later phases of fibrosis and remodeling [139]. TLR4^*−/−*^ mice are more susceptible to intratracheal bleomycin-induced lung fibrosis due to (1) impaired type 2 alveolar epithelial cells renewal, which are critical cells in the fibrosis repair process [162], and (2) impaired activation of autophagy signaling leading to accumulation of ROS [139].

Although we focused on macrophages and their interactions with fibroblasts in this review, a wide range of immune cell types are also involved in the progression and resolution of fibrosis [163]. Neutrophils are the cells that are recruited early stages of the fibrotic process, mice depleted from neutrophils have ameliorated response to, and the failure in recruiting neutrophils protects mice from bleomycin-induced pulmonary fibrosis [164, 165]. On the other hand, natural killer cells may have a protective effect against lung fibrosis [166]. Without NK cell recruitment, the pulmonary environment lacks IFN-γ, an important anti-inflammatory cytokine involved in the resolution of fibrosis [167]. This results in an enhanced fibrosis process in the lung [168, 169]. Dendritic cells (DCs), however, may play a dual role in pulmonary fibrosis. Like neutrophils, DCs arrive in the early phases of pulmonary fibrosis in significant numbers, and inhibiting the immune activity of DCs attenuates fibrosis [170]. However, it has also been observed that mice deficient in DCs develop more severe fibrosis, and, in contrast, mice equipped with an increasing number of DCs develop milder pulmonary fibrosis after the bleomycin challenge [171]. The mechanisms by which DCs exert their pro/antifibrotic role remain to be further elucidated [172]. We believe macrophages are the most pertinent to this review because they are the master regulator of fibrosis across organs, given that they are the primary providers of TGF-β [173]. Additionally, the close interaction of macrophages with fibroblasts is a critical contributor to the cycle described in this review [174, 175].

In this review, we summarized the current state of knowledge regarding the role of DAMPs, selected proinflammatory cytokines, their interplay through TLRs (more specifically TLR4), and their contribution to cellular processes of lung fibrosis. Furthermore, we highlighted knowledge gaps and summarized the therapeutic potential of targeting this vicious fibrotic cycle at every checkpoint. Given that the issue of persistent fibrosis without resolution in COVID-19, IPF, and other profibrotic lung diseases is far from resolved, it is critical to look deeper into these pathways to illuminate not only the connection between the inflammatory reaction and fibrosis but also develop possible therapeutics that can ameliorate pulmonary fibrosis by disrupting the positive feedback pathways involved.
